# Characterization of Insecticide Response-Associated Transcripts in the Colorado Potato Beetle: Relevance of Selected Cytochrome P450s and Clothianidin

**DOI:** 10.3390/insects13060505

**Published:** 2022-05-26

**Authors:** Raed Bouafoura, Pierre Bastarache, Brigitte Christelle Ouédraogo, Pascal Dumas, Chandra E. Moffat, Jess L. Vickruck, Pier Jr Morin

**Affiliations:** 1 Department of Chemistry and Biochemistry, Université de Moncton, 18 Antonine-Maillet Avenue, Moncton, NB E1A 3E9, Canada; erb8993@umoncton.ca (R.B.); epb6696@umoncton.ca (P.B.); ebo3875@umoncton.ca (B.C.O.); epd3362@umoncton.ca (P.D.); 2 Fredericton Research and Development Centre, Agriculture and Agri-Food Canada, 95 Innovation Road, Fredericton, NB E3B 4Z7, Canada; chandra.moffat@agr.gc.ca (C.E.M.); jess.vickruck@agr.gc.ca (J.L.V.)

**Keywords:** Colorado potato beetles, clothianidin, chlorantraniliprole, imidacloprid, spinosad, cytochrome P450s, glutathione s-transferases, cuticular proteins

## Abstract

**Simple Summary:**

The Colorado potato beetle is an insect pest that can significantly harm potato crops. Various approaches are available to mitigate its damages including the use of insecticides. Unfortunately, its ability to develop resistance towards these compounds is substantial, and understanding the basis of this process is of utmost importance to design strategies to limit the impact of this insect. This work thus aims at quantifying the expression of key transcripts coding for proteins associated with insecticide resistance in Colorado potato beetles exposed to four insecticides. Significant variations were observed, notably in insects exposed to the insecticide clothianidin. Interestingly, subsequent reduction of endogenous levels of selected targets modulated by clothianidin was associated with increased insect susceptibility to this neonicotinoid. These results further highlight molecular players with potential relevance for insecticide resistance, and introduce novel targets that underlie clothianidin resistance in the Colorado potato beetle.

**Abstract:**

The Colorado potato beetle (*Leptinotarsa decemlineata* (Say)) is known for its capacity to cause significant damages to potato crops worldwide. Multiple approaches have been considered to limit its spread including the use of a diverse arsenal of insecticides. Unfortunately, this insect frequently develops resistance towards these compounds. Investigating the molecular bases underlying the response of *L. decemlineata* against insecticides is of strong interest to ultimately devise novel and targeted approaches aimed at this pest. This work aimed to characterize, via qRT-PCR, the expression status of targets with relevance to insecticide response, including ones coding for cytochrome P450s, glutathione s-transferases, and cuticular proteins, in *L. decemlineata* exposed to four insecticides; chlorantraniliprole, clothianidin, imidacloprid, and spinosad. Modulation of levels associated with transcripts coding for selected cytochrome P450s was reported in insects treated with three of the four insecticides studied. Clothianidin treatment yielded the most variations in transcript levels, leading to significant changes in transcripts coding for *CYP4c1*, *CYP4g15*, *CYP6a13*, *CYP9e2*, *GST*, and *GST-1-Like*. Injection of dsRNA targeting *CYP4c1* and *CYP9e2* was associated with a substantial decrease in expression levels and was, in the case of the latter target, linked to a greater susceptibility of *L. decemlineata* towards this neonicotinoid, supporting a potential role for this target in clothianidin response. Overall, this data further highlights the differential expression of transcripts with potential relevance in insecticide response, as well as generating specific targets that warrant investigation as novel dsRNA-based approaches are developed against this insect pest.

## 1. Introduction

The Colorado potato beetle, *Leptinotarsa decemlineata* (Say), is an insect pest that can be considered a primary threat to potato plants in several geographical regions of the world [1]. The potential damages associated with this pest on potato plants can be highlighted by early work reporting consumption of 40 cm^2^ of potato leaves by one *L. decemlineata* throughout its larval stage [2]. Defoliation can lead to losses in yield of up to 80% in impacted potato fields [3]. A plethora of strategies have been explored to control the damages and spread of this insect, including cultural practices that leverage crop rotation [4] and biological approaches that rely on natural enemies such as the spotted lady beetle *Coleomegilla maculata* and the two-spotted stink bug *Perillus bioculatus*, to name a few [5,6,7]. The primary option to control this insect pest nevertheless involves the use of insecticides from various classes, including neonicotinoids, such as imidacloprid and thiamethoxam, and diamides, such as chlorantraniliprole. While these compounds have contributed to controlling *L. decemlineata* with various levels of success, resistance against insecticides has been reported in this insect and remains an important challenge in management of this potato pest [8,9].

Much interest has thus been placed in recent years in characterizing the underlying molecular bases associated with insecticide response and resistance in *L. decemlineata*. Several targets have emerged for their relevance with insecticides susceptibility in this insect. Early work conducted in cyhalothrin-treated *L. decemlineata* larvae notably revealed upregulation of multiple transcripts coding for cytochrome P450s such as *Cyp12H2*, *Cyp6BH2*, *Cyp6BJ1*, *Cyp6BQ17*, and *Cyp6EG1*, to name a few [10]. Transcriptomics-based work performed in adult *L. decemlineata* revealed three upregulated transcripts coding for cytochrome P450s, including *CYP9z4*, in numerous populations that displayed resistance towards imidacloprid [11]. Subsequent work revealed that RNA interference (RNAi)-based downregulation of a cytochrome P450, as well as of a cuticular protein and a glutathione synthetase, was associated with increased sensitivity to this compound [12]. Targeting of transcripts coding for a cuticular protein and glutathione synthetase via double-stranded RNA (dsRNA) notably revealed synergism between this approach and treatments with a decreased dose of imidacloprid in 2nd instar *L. decemlineata* larvae [13]. A group of four transcripts coding for glutathione s-transferases, such as *LdGSTe2a*, *LdGSTe2b*, *LdGSTo5*, and *LdGSTt1* also displayed elevated levels following treatment of *L. decemlineata* with cyhalothrin, fipronil, or endosulfan [14]. While additional work has also leveraged high-throughput transcriptomics-based approaches to reveal signatures of response associated with selected insecticides, including chlorothalonil, imidacloprid, and spinosad [15,16], characterization of modulated transcripts in *L. decemlineata* in response to insecticide exposure remains of utmost interest.

This study was initiated to characterize the expression profile of transcript targets coding for proteins linked to families known for their potential role in insecticide response, including cytochrome P450s, glutathione s-transferases, and cuticular proteins, in Colorado potato beetles exposed to four insecticides. Knockdown of selected transcripts was undertaken using RNAi to assess their underlying impact on response to insecticides that displayed the strongest modulation of the studied targets. This work presents varying levels of several transcripts following insecticide treatments in *L. decemlineata* and warrants their ongoing investigation as targets of interest in this potato pest.

## 2. Materials and Methods

### 2.1. Insects

Colorado potato beetles *Leptinotarsa decemlineata* (Say) were sourced in the summers of 2017 and 2018 via collaborators at the Fredericton Research and Development Centre (45°55′17.5″ N 66°36′01.8″ W, Fredericton, NB, Canada). Insects were initially sampled from fields not previously treated with insecticides. Adult insects were brought to Moncton (NB, Canada) in plastic containers in which potato leaves (var. Kennebec) were added as a food source. Upon arrival, insects were acclimated for no less than five days at 25 °C using an incubator (Thermo Fisher Scientific, Waltham, MA, USA). Insects were maintained in a 16L:8D photoperiod throughout the experiment. Exposure to the four insecticides of interest, chlorantraniliprole, clothianidin, imidacloprid, and spinosad, was conducted in four separate time courses. Treatment with chlorantraniliprole was performed as presented previously [17]. A volume of 1 μL (1 µg) of a chlorantraniliprole solution (#32510, Sigma-Aldrich, St. Louis, MO, USA), prepared in acetone, was pipetted topically on the abdomen of adult *L. decemlineata*. An equal volume of acetone was applied to insects that served as controls. Insects were placed in the incubator for a period of 24 h after which the insects were frozen in liquid nitrogen. Clothianidin exposure was completed by topical application of a volume of 1.25 μL (0.25 µg) of clothianidin (#33589, Sigma-Aldrich), dissolved in acetone, on the abdomen of adult insects. A similar volume of acetone was applied to insects that were used as controls. Insects were returned to an incubator for 4 h and sampling was performed as above. Time course for exposure to imidacloprid was performed as before [18]. Topical application of a volume of 5 μL (0.5 µg) of an imidacloprid solution in acetonitrile (#46341, Sigma-Aldrich) was performed on the abdomen of adult insects. A group of insects serving as controls were exposed with an equal volume of acetonitrile. Acetonitrile was used for the imidacloprid exposure time course specifically considering it was the solvent in which the insecticide was provided by the supplier. An incubation period of 24 h ensued, followed by storage in liquid nitrogen. Adult insects were exposed to spinosad using a protocol conducted previously [16]. Topical application of 0.5 µL (0.5 µg) of a spinosad solution (#33706, Sigma-Aldrich), prepared in acetone, was performed on the abdomen of insects. A comparable amount of acetone was deposited on the abdomen of insects used as controls. Insects were subsequently returned to the incubator for 4 h and sampled as above.

### 2.2. RNA Isolation

RNA isolates were prepared from *L. decemlineata* exposed to different insecticides and from control insects as described before [17]. The miRVana miRNA Isolation Kit (Thermo Fisher Scientific) was used to isolate large RNA fractions following the manufacturer’s protocol. Replicates each contained two insects as starting material. RNA concentrations of all isolates were measured using the NanoVue Plus Spectrophotometer (Thermo Fisher Scientific). RNA fractions were placed at −80 °C until use.

### 2.3. Synthesis of cDNA

The synthesis of cDNA for transcripts amplification and quantification was conducted with 1 µg of starting RNA. This amount was combined with 1 µL oligo dT, 1 µL 10 mM dNTPs, and DEPC-treated water to a final volume of 12 µL. Mixture was incubated at 65 °C for 5 min. Reagents consisting of 4 µL 5× First Strand Buffer, 2 µL 0.1 M DTT, and 1.5 µL DEPC-treated water were added next. This solution was incubated at 37 °C for 2 min followed by addition of 0.5 µL M-MLV RT. A final incubation period at 37 °C for 50 min and at 70 °C for 15 min was performed.

### 2.4. qRT-PCR Amplification of Transcripts of Interest

Transcripts were quantified using qRT-PCR. Primers for amplification of cytochrome P450 4c1-like (*CYP4c1*; LOC111503441), cytochrome P450 4g15-like (*CYP4g15*; LOC111502305), cytochrome P450 6a13 (*CYP6a13*; LOC111510690), cytochrome P450 6a23 (*CYP6a23*; LOC111517755), cytochrome P450 9e2-like (*CYP9e2*; LOC111507098), cytochrome P450 12a5 mitochondrial (*CYP12a5*; LOC111510459), glutathione S-transferase C-terminal domain-containing protein (*GST*; LOC111513254), glutathione S-transferase 1 (*GST-1*; LOC111515122), glutathione S-transferase 1-like (*GST-1-Like*; LOC111509100), cuticle protein 1-like (*CP-1*; LOC111505597), and uncharacterized cuticle protein (*CP-141*; LOC111512529) in *L. decemlineata* were conceived and are presented in Table 1. Initial reactions consisted of 5 µL of a cDNA template diluted by a factor of 10, 1 µL 25 µM forward primer, 1 µL 25 µM reverse primer, 5.5 µL DEPC-treated water, and 12.5 µL 2× Taq FroggaMix. Protocol undertaken for amplification comprised of an initial step at 95 °C for 5 min, followed by 40 cycles at 95 °C for 15 s, at temperature gradient between 50 and 65 °C for 30 s and 72 °C for 45 s. Products were detected on a 2% agarose gel and further sequenced using the Université Laval sequencing services (Quebec City, QC, Canada). Transcript targets were also amplified using a range of annealing temperatures and on serial cDNA dilutions to evaluate efficiency of each primer pair. Target quantification was performed by mixing 2.5 µL of cDNA template (10^−1^), 0.5 µL DEPC-treated water, 1 µL 5 µM forward primer, 1 µL 5 µM reverse primer, and 5 µL of iTaq Universal SYBR Green Supermix (Bio-Rad, Hercules, CA, USA). Protocol consisted of an initial step at 95 °C for 3 min, followed by 40 cycles at 95 °C for 15 s and at the identified optimal annealing temperature for 30 s. Levels of *RP-18*, amplified in parallel reactions, were used as reference. An unpaired Student’s *t*-test was conducted to assess significant differences between control and insecticide-exposed insects. Normalized transcript expression and statistical analysis were performed using the Bio-Rad CFX Maestro software (v1.1).

### 2.5. dsRNA Synthesis

Synthesis of dsRNA was performed using the MEGAscript RNAi Kit (Thermo Fisher Scientific) following manufacturer’s instructions. Fragments coding for *CYP4c1* and *CYP9e2* were chosen as RNAi target-sequences from *L. decemlineata* sequences. T7 primers for *CYP4c1*-targeting dsRNA synthesis were forward 5′-TAATACGACTCACTATAGGGAGAAATCCCTGTCAATGGCAGAC-3′ and reverse 5′-TAATACGACTCACTATAGGGAGATCAGGAGCGAGCTTCAACTT-3′. T7 primers for CYP9e2-targeting dsRNA synthesis were forward 5′-TAATACGACTCACTATAGGGAGACGTGAGTTGGTGGATGACA-3′ and reverse 5′-TAATACGACTCACTATAGGGAGACGGTCCGGATCTGGATAGTA-3′. PCR amplification of the target fragments was performed at 95 °C for 5 min, followed by 40 cycles of 95 °C for 15 s, at 51.9 °C for the former and 55.0 °C for the latter for 30 s, 72 °C for 45 s, followed by a final step of 72 °C for 5 min. PCR products were purified using the QIAquick PCR Purification Kit (QIAGEN, Hilden, Germany) and sequenced as above. dsRNAs were produced using PCR products as templates and treated subsequently with DNase and RNase. Purified dsRNA was assessed on a 2% agarose gel and quantified on a NanoVue Plus Spectrophotometer. Products were stored at −20 °C until injection.

### 2.6. dsRNA Injection

dsRNA was injected in *L. decemlineata* using a 10 µL Hamilton Microliter Syringe (Hamilton, Reno, NV, USA). Insects were immobilized on their back using plasticine. Insects were injected in the abdomen with 5 µL of a 500 ng/µL dsRNA solution in the case of *CYP4c1* (*n* = 30) or with 5 µL of a 600 ng/µL dsRNA solution in the case of *CYP9e2* (*n* = 30). Control insects were injected with an equal volume of saline solution used to dilute dsRNAs (*n* = 30). Insects were kept in an incubator (Thermo Fisher Scientific) and held under a 16L:8D photoperiod at 25 °C for the duration of the experiment, which was performed once.

### 2.7. Transcript Targets Silencing Assessment

Target knockdown efficiency was assessed by qRT-PCR in *L. decemlineata* seven days following injection with saline or dsRNA targeting *CYP4c1* and *CYP9e2*. RNA isolation was conducted as described above, with the difference that total RNA was isolated with Trizol (Thermo Fisher Scientific) following manufacturer’s protocol and using one insect per replicate (*n* = 4). Primers to evaluate knockdown efficiency of targets were designed outside of the region covered by the dsRNA sequences and conceived towards the 5′ end of the region targeted by the dsRNA. Primers to quantify *CYP4c1* and *CYP9e2* transcript levels are presented in Table 1. Quantification by qRT-PCR was performed at 95 °C for 3 min, followed by 45 cycles of 95 °C for 15 s and at optimal annealing temperature for 30 s. Transcript levels of RP-18 were measured in the same samples and used as reference. Significant differences between target expression were evaluated using an unpaired Student’s *t*-test.

### 2.8. Clothianidin Treatment in Insects Injected with dsRNA

The impact of clothianidin treatment following dsRNA-mediated modulation of *CYP4c1* and *CYP9e2* expression levels was assessed in three groups (*n* = 26) comprising saline-, dsRNA *CYP4c1*-, or dsRNA *CYP9e2*-injected *L. decemlineata* placed individually in Petri dishes. Seven days following the injection, a number of insects (*n* = 13) from each group were separated and exposed to 1.25 µL of a 0.2 µg/µL clothianidin solution prepared in acetone, corresponding to a dose of 0.25 µg of clothianidin, and the rest of the insects (*n* = 13) from each group were treated with 1.25 µL of acetone. This approach to investigate the impact of clothianidin exposure in dsRNA-injected insects using this dose of insecticide was performed once. Clothianidin exposure tests had been conducted initially in *L. decemlineata* using 0.1 µg, 0.4 µg, 1.0 µg, 2.5 µg, 5.0 µg, 10.0 µg, and 20.0 µg of clothianidin. Significant impact was observed after 24 h at the five highest doses (Table S1). Insect mortality, evaluated following mild agitation of dish and by scoring for the ability of insect to right itself as published previously [19], was performed following clothianidin exposure.

### 2.9. Quantification and Statistical Analysis

Acquisition of quantification cycles through qRT-PCR was performed using Bio-Rad CFX Manager. Relative normalized transcript levels and statistical analysis were accomplished via the CFX Maestro software (v1.1, Bio-Rad Laboratories, Portland, ME, USA). A Grubb’s test was conducted to identify any statistical outlier. Differences in transcript level expression between control insects and insects exposed to insecticides were assessed using an unpaired Student’s *t*-test.

## 3. Results

### 3.1. Transcript Expression in Chlorantraniliprole-Exposed L. decemlineata

**Figure 1:**
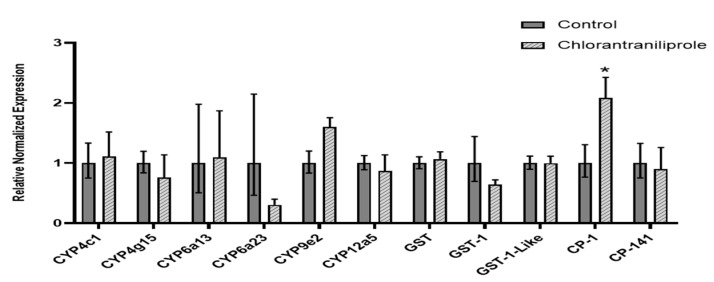
Transcript levels, normalized to *RP-18*, in chlorantraniliprole-exposed versus control insects. Data are mean standardized transcript levels (mean ± SEM, *n* = 4–5). Asterisks represent results significantly different from control samples (* *p* < 0.05).

Levels of transcripts coding for *CYP4c1*, *CYP4g5*, *CYP6a13*, *CYP6a23*, *CYP9e2*, *CYP12a5*, *GST*, *GST-1*, *GST-1-Like*, *CP-1*, and *CP-141* were quantified by qRT-PCR in *L. decemlineata* submitted to chlorantraniliprole treatments (Figure 1). Expression status remained stable for most of the targets assessed. Significant upregulation of 2.08-fold (*p* < 0.05) was observed for *CP-1* transcript levels in insects exposed to chlorantraniliprole versus control insects. *CYP6a23* and *GST-1* transcript levels were downregulated, albeit non-significantly, to levels that were 0.33-fold and 0.64-fold the ones measured in control *L. decemlineata*.

### 3.2. Transcript Expression in Clothianidin-Exposed L. decemlineata

**Figure 2:**
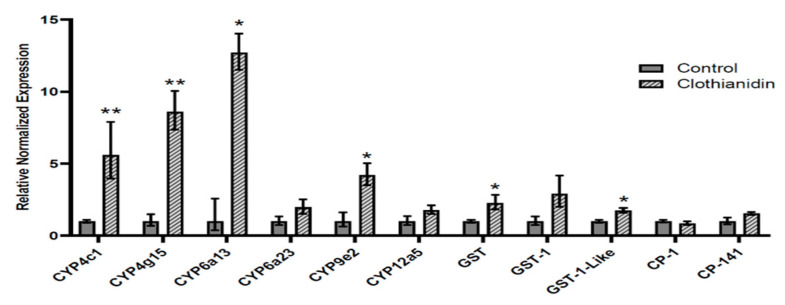
Transcript levels, normalized to *RP-18*, in insects submitted to clothianidin versus control insects. Data are mean standardized transcript levels (mean ± SEM, *n* = 4–5). Asterisks depict results that are significantly different (* *p* < 0.05, ** *p* < 0.01).

Expression status of targets of interest was measured in insects treated with clothianidin using qRT-PCR (Figure 2). Multiple transcript targets, six out of the eleven studied, displayed elevated levels in insecticide-exposed *L. decemlineata* when compared with control insects, including *CYP4c1*, *CYP4g15*, *CYP6a13*, *CYP9e2*, *GST*, and *GST-1-Like,* with changes of 5.60-fold (*p* < 0.01), 8.60-fold (*p* < 0.01), 12.71-fold (*p* < 0.05), 4.20-fold (*p* < 0.05), 2.27-fold (*p* < 0.05), and 1.75-fold (*p* < 0.05), respectively.

### 3.3. Transcript Expression in Imidacloprid-Exposed L. decemlineata

**Figure 3:**
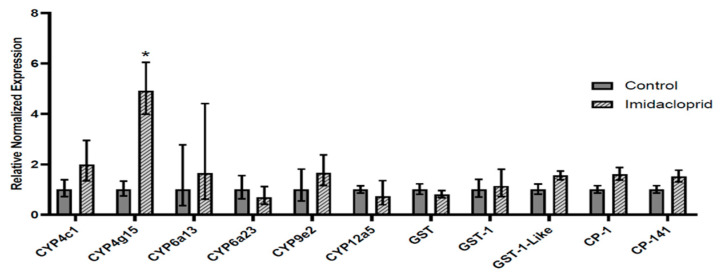
Expression of transcript levels, normalized to *RP-18*, in imidacloprid-treated versus untreated insects. Data are mean standardized transcript levels (mean ± SEM, *n* = 3). Asterisks depict results that are significantly different (* *p* < 0.05).

Levels of transcripts of interest were assessed in *L. decemlineata* exposed to imidacloprid and compared with expression observed in control insects (Figure 3). The strongest modulation in transcript levels was observed for *CYP4g15*, which exhibited a 4.92-fold (*p* < 0.05) change in insects treated with this neonicotinoid versus insects that were used as controls. Transcript levels of *CYP4c1* were elevated, albeit not significantly, to levels that were 1.99-fold the levels observed in insects used as controls.

### 3.4. Transcript Expression in Spinosad-Exposed L. decemlineata

**Figure 4:**
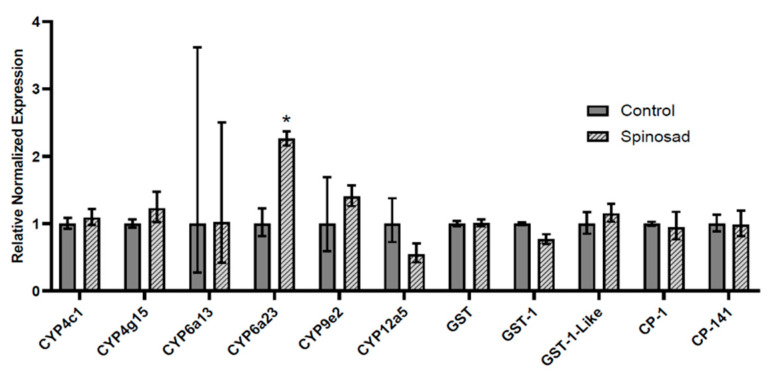
Expression of transcript levels, normalized to *RP-18*, in spinosad-treated versus untreated insects. Data are mean standardized transcript levels (mean ± SEM, *n* = 4–5). Asterisks represent results significantly different from control samples (* *p* < 0.05).

Insects were treated with spinosad and levels of transcripts of interest were subsequently measured by qRT-PCR (Figure 4). Transcript levels remained stable for most of the measured targets. Transcript levels of *CYP6a23* displayed changes of 2.26-fold (*p* < 0.05) in *L. decemlineata* submitted to spinosad versus control insects.

### 3.5. Mortality of L. decemlineata in dsRNA-Injected Insects Exposed to Clothianidin

**Figure 5:**
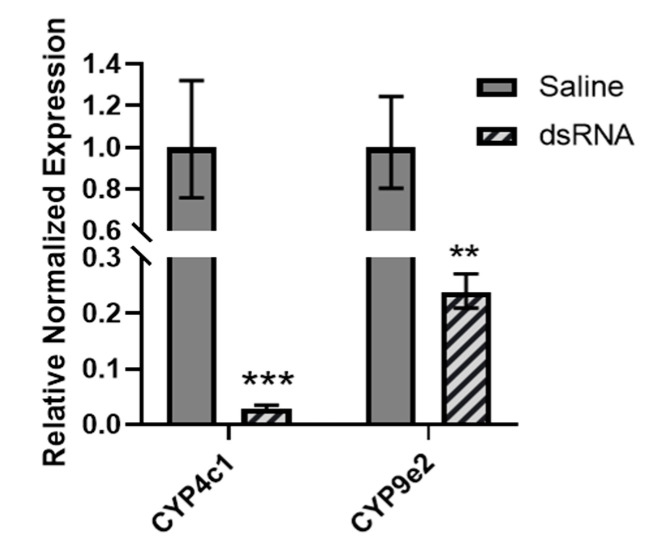
RNAi-based downregulation of *CYP4c1* and *CYP9e2* in saline- and dsRNA-injected *L. decemlineata*. Histogram presents expression levels of targets of interest in dsRNA-injected insects seven days following injection quantified by qRT-PCR. Controls used were insects injected with saline solution. Results shown are standardized transcript levels (mean ± SEM, *n* = 4). Asterisks present results that are significantly different (** *p* < 0.01, *** *p* < 0.001).

Expression status in insects exposed to four insecticides: chlorantraniliprole, clothianidin, imidacloprid and spinosad, revealed that clothianidin elicited the strongest modulation of transcript targets with a total of six out of the eleven investigated that were differentially expressed (54.5%). Further work was thus undertaken to assess the impact of modulating selected targets of interest on clothianidin response in *L. decemlineata*. Knockdown of *CYP4c1* and *CYP9e2* was performed using a dsRNA-based approach. Knockdown efficiency was confirmed for all targets investigated using qRT-PCR (Figure 5). Transcript levels of *CYP4c1* were reduced to levels that were 0.03-fold (*p* < 0.001) the values observed in insects used as controls. Similarly, transcript levels of *CYP9e2* were decreased to 0.24-fold (*p* < 0.01) the ones measured in saline-injected insects.

**Figure 6:**
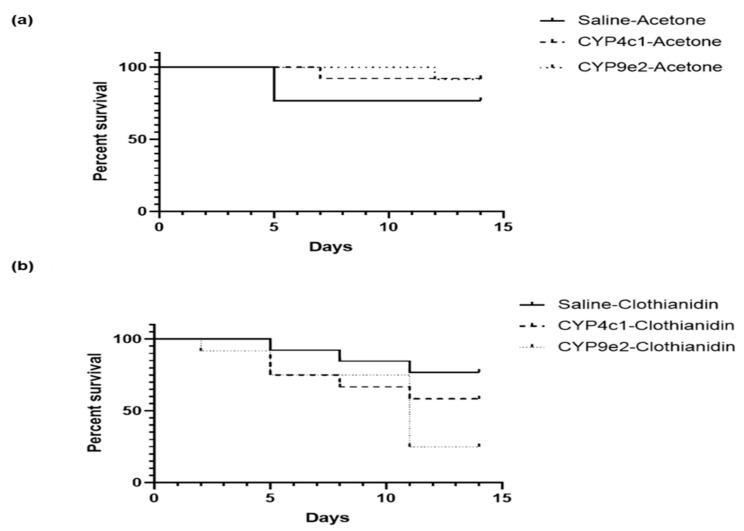
Effect of clothianidin treatment on *L. decemlineata* in control and dsRNA-injected insects. Kaplan-Meier survival analysis depicts *L. decemlineata* mortality in saline- or dsRNA-injected insects treated with 0.25 µg of clothianidin. (**a**) Survival analysis depicting the impact of *CYP4c1* and *CYP9e2* modulation in insects treated with acetone (*n* = 12–13). (**b**) Survival analysis depicting the effect of *CYP4c1* and *CYP9e2* variation in insects exposed to clothianidin (*n* = 12–13).

Insect survival was subsequently monitored in dsRNA- or saline-injected insects submitted to a clothianidin challenge. No significant change was observed between saline-, dsRNA *CYP4c1*-, and dsRNA *CYP9e2*-injected insects when acetone treatment was performed (Figure 6a). Changes were, on the other hand, observed in insects injected with saline or dsRNAs and subsequently exposed to clothianidin seven days following injection. Differences in insect mortality, albeit not significant, were observed in clothianidin-treated insects that had been injected with dsRNA targeting *CYP4c1* when compared with insects that had been injected with saline. In addition, significant changes were measured in clothianidin-exposed *L. decemlineata* that had been injected with dsRNA aimed at *CYP9e2* when compared with saline-injected insects with percentage of deceased insects at 75.0% and 23.1%, respectively (Log-rank test *p* = 0.02) (Figure 6b).

## 4. Discussion

The current study was initiated to explore the differential expression of targets with relevance to insecticide response and resistance in *L. decemlineata*. A focus was placed on the quantification of selected transcripts coding for cytochrome P450s, cuticular proteins and glutathione s-transferases in insects treated with compounds including the neonicotinoid imidacloprid or the carboxamide chlorantraniliprole. Results showed several deregulated transcripts following exposure to selected insecticides, and identified targets with potential involvement in the molecular response associated with selected insecticide response in this potato pest.

Multiple reports have investigated, in recent years, the molecular changes associated with response to treatments with imidacloprid in various insects including *L. decemlineata*. Pioneering work on this topic highlighted different signatures of genes, including certain coding for cytochrome P450 proteins, in *L. decemlineata* populations that were resistant versus susceptible to imidacloprid [11]. Similar upregulation in the expression of selected cytochrome P450s was also reported in *L. decemlineata* resistant to imidacloprid when compared with insects that were susceptible to this compound [20]. Follow-up work further revealed the potential importance of cap ‘n’ collar isoform C (*CncC*), a transcription factor that can drive the expression of various cytochrome P450s, for imidacloprid response in this insect pest [21]. Expression levels of transcripts coding for cytochrome P450s and cuticular proteins were substantially elevated in *L. decemlineata*, and this increase was linked with peak resistance to imidacloprid [12]. It is interesting to note that downregulation of selected transcripts coding for cuticular proteins via a dsRNA approach substantially decreased *L. decemlineata* larvae survival, and that combination of small doses of these dsRNAs with imidacloprid contributed to a synergistic effect in this insect [13]. The current work notably revealed the significant upregulation of a transcript coding for cytochrome P450 *CYP4g15* in adult *L. decemlineata* exposed to imidacloprid. This target has been previously reported as over-expressed in an imidacloprid-resistant strain of the Asian citrus psyllid, *Diaphorina citri* [22]. Significant elevation of transcripts coding for *CYP4g15* was also reported in the mosquito *Aedes aegypti* that was deemed resistant to multiple insecticides including permethrin and malathion [23]. Modulation of *CYP4g15* was also observed in the aquatic dipteran *Prodiamesa olivacea* following exposure to various xenobiotics [24]. These studies, combined with the current data, support the potential significance of *CYP4g15* in response to insecticides. A closer examination regarding its function in imidacloprid response in *L. decemlineata* is warranted.

Modulation of the transcripts of interest was next explored in *L. decemlineata* exposed to insecticides, such as chlorantraniliprole, spinosad, and clothianidin, for which information regarding their molecular impacts in this insect was sparse. Recent work aligned with this theme has nevertheless shed light on selected molecular targets modulated by insecticides in *L. decemlineata*. A proteomics-based approach conducted in *L. decemlineata* resistant or susceptible to various insecticides notably showed upregulation of a C-type lectin [25]. Early work conducted on chlorantraniliprole in *L. decemlineata* demonstrated that dsRNA-based modulation of a target coding for a ryanodine receptor had an impact on its response towards this compound [26]. A qRT-PCR-based approached reported differential expression of transcripts coding for cytochrome P450s and of microRNA transcripts in *L. decemlineata* exposed to this compound [17]. The current study revealed a significant upregulation of a transcript coding for the cuticular protein *CP-1*. Previous work has notably revealed a common over-expression of several transcripts coding for cuticular proteins in the diamondback moth *Plutella xylostella* treated with five insecticides including chlorantraniliprole and spinosad, supporting a relevance for these targets in insecticide response [27]. Regarding the latter compound, against which substantial field-evolved resistance has been reported for *L. decemlineata* notably from organically managed fields [28], recent work has identified via next-generation sequencing a total of 30 deregulated mRNA transcripts in *L. decemlineata* submitted to spinosad treatments [16]. Downregulation of a transcript coding for Mesh yielded a similar efficacy when compared with spinosad in field trials aimed at *L. decemlineata* larvae [29]. Data presented here showed upregulation of *CYP6a23* transcript levels in insects exposed to spinosad when compared with control insects. Not much is known regarding the significance of this target with respect to insecticide response in insects. Changes in deltamethrin resistance were linked to mutations in the fruit fly *Drosophila melanogaster* [30]. Early work in the same model had nonetheless observed no difference in survival in flies with increased levels of *CYP6a23* and exposed to four different insecticides [31]. Interestingly, the work presented here highlighted the greatest number of deregulated transcripts in insects that had been treated with clothianidin. Transcripts coding for *CYP4c1*, *CYP4g15*, *CYP6a13*, *CYP9e2*, and two glutathione s-transferases were upregulated in clothianidin-treated insects. Information is sparse regarding the molecular response to this compound in this insect, while studies have been conducted in other insects. A total of 12 transcripts coding for cytochrome P450 genes were upregulated in a strain of the brown planthopper *Nilaparvata lugens* that showed resistance to several compounds, with the most affected being nitenpyram and clothianidin [32]. Induction of cytochrome P450 activities was reported in the honeybee *Apis mellifera* treated with clothianidin [33]. Several transcripts associated with cytochrome P450s and glutathione s-transferases were shown to be modulated notably following clothianidin exposure in the leek maggot *Bradysia odoriphaga* [34]. Besides transcriptional changes with relevance to clothianidin, expression status of *CYP6a13* also displayed a positive correlation with pyrethroid resistance in different populations of the common cutworm, *Spodoptera litura*, that were identified as pyrethroid-resistant [35]. In addition, overexpression of *CYP12a5* in *D. melanogaster* resulted in greater sensitivity to the compound nitenpyram [36]. The present work was aligned with these findings by reporting differential expression of transcripts coding for cytochrome P450s and glutathione s-transferases in *L. decemlineata*. Injection of dsRNA targeting two such transcripts, coding for *CYP4c1* and *CYP9e2*, were investigated in this study. Besides *CYP4c1* and *CYP9e2* overexpression observed in *L. decemlineata* exposed to clothianidin, previous work revealed a potential relevance for these targets with respect to insecticide resistance in insects exposed to various compounds, further supporting their potential importance in insecticide management [37,38]. Adult *L. decemlineata* injected with dsRNA targeting the latter notably revealed significant differences in insects treated with clothianidin when compared with insects used as controls, suggesting an underlying function for this transcript with respect to clothianidin response in this insect. Additional work, including similar treatments with reduced doses of clothianidin or with *CYP9e2*-targeting dsRNA feeding bioassays, is envisioned to better asses the role of *CYP9e2* in *L. decemlineata* insecticide response. It is also important to note that, while the data presented in the current work provide valuable information on deregulated transcripts in response to insecticides in *L. decemlineata* not previously exposed to various compounds, subsequent efforts are warranted to better delineate the underlying roles, if any, of these targets in the process of insecticide resistance. Characterizing the expression status of these transcripts in *L. decemlineata* populations with confirmed resistance towards a given compound of interest such as clothianidin, similar to work conducted on the potential role of *CYP6er1* in clothianidin-resistant populations of *N. lugens* [39], would be an interesting starting point to better position these targets within the context of insecticide resistance in this potato pest. It is finally important to point out that leveraging, in the mid- to long-term, a high-throughput sequencing analysis approach to decipher a plethora of deregulated transcripts, besides the select group assessed in the current work, would yield valuable information and should be considered.

## 5. Conclusions

In conclusion, this work explored the expression status of multiple transcript targets with relevance to insecticide response in *L. decemlineata*. Selected transcripts coding for cytochrome P450s, glutathione s-transferases, or cuticular proteins displayed significant upregulation in insects exposed to insecticides when compared with control insects. *CYP4g15* transcript levels were notably elevated in insects treated with the neonicotinoids clothianidin and imidacloprid. Variations in expression status of several cytochrome P450s: *CYP4c1*, *CYP4g15*, *CYP6a13*, and *CYP9e2*, were observed in clothianidin-exposed insects. Subsequent dsRNA-based modulation of two cytochrome P450s was performed in insects treated with clothianidin, and *CYP9e2* knockdown was associated with increased susceptibility to this compound, thus presenting a target to further explore in the investigation of RNAi-based approaches to manage *L. decemlineata*. Additional functional work aimed at characterizing the effects of dsRNA-based variation of specific transcripts, besides *CYP4c1* and *CYP9e2*, that displayed changes in expression following treatment with selected insecticides, in addition to clothianidin, is a perspective to consider in order to build on the information presented in this study. Quantification of the transcripts of interest investigated here in a greater number of insects, or evaluation of the response of these targets in insects exposed to other compounds relevant to Colorado potato beetle management and resistance such as thiamethoxam [40], would also contribute to further strengthening the preliminary expression profiles generated in this project. A more thorough investigation of the level of resistance associated with the insecticides of interest, via bioassays in a greater number of insects, should be considered. Overall, this work is aligned with ongoing efforts to better understand the molecular bases underlying insecticide response in *L. decemlineata* and adds to the growing number of molecular leads of interest with potential relevance to insecticide management in this notorious potato pest.

## Figures and Tables

**Table 1:** Primers used for quantification by qRT-PCR of relevant transcripts. Abbreviations: Forward (Fwd) and Reverse (Rev) primers.

Primer		Sequence	Efficiency	Temperature
*CYP4c1*	Fwd Rev	5′-TGGCTTGGATTGGGATTGCT-3′ 5′-CCATTGCGGCCTCACAAATT-3′	102.5%	60.2 °C
*CYP4g15*	Fwd Rev	5′-TGTCGGAGCAAGTGGTGATT-3′ 5′-GGTGACTTTCCGATGCCCAT-3′	91.7%	60.2 °C
*CYP6a13*	Fwd Rev	5′-ACCTTAGTGGCCTGCACTTC-3′ 5′-GGATTCCCCTGAGTTTTCCGA-3′	103.6%	60.2 °C
*CYP6a23*	Fwd Rev	5′-CCGTTTGCCATTTTGTGTGG-3′ 5′-TGGCTGCAAGTTTCCCCAT-3′	96.4%	60.2 °C
*CYP9e2*	Fwd Rev	5′-TCGTTCCCAAGTCCTTGCAA-3′ 5′-CCAACTGTCCCCAAAAAGCC-3′	93.3%	60.2 °C
*CYP12a5*	Fwd Rev	5′-GAGTCTAAGCCTCCCGGTTG-3′ 5′-ATACGTAATCTGACCGCCCG-3′	86.7%	57.1 °C
*GST*	Fwd Rev	5′-TGGCTTGTGTTCCGTAGCAA-3′ 5′-ATCGCCTGGCAAGCAAAATC-3′	95.6%	53.5 °C
*GST-1*	Fwd Rev	5′-CTGTCTCACTCGAACGCAGA-3′ 5′-TGGCGGTCCATCTGATACAG-3′	97.4%	62.8 °C
*GST-1-Like*	Fwd Rev	5′-GCTCAAGGCTTACCAGATGC-3′ 5′-CGTTCAGATGGGCCCTTCTT-3′	85.4%	62.8 °C
*CP-1*	Fwd Rev	5′-ATTTTATCGGCGCTCTTGGC-3′ 5′-TGCGTTTCGACCTTCCTTCA-3′	109.7%	60.2 °C
*CP-141*	Fwd Rev	5′-CTCCCACCACGTAAGGAAGA-3′ 5′-AGTTGGGTTCCTGGCTCTCT-3′	107.7%	58.3 °C
*RP-18*	Fwd Rev	5′-TAGAATCCTCAAAGCAGGTGGCGA-3′ 5′-AGCTGGACCACCGTGTTTCACTGC-3′	110.1%	60.0 °C

## Data Availability

The data presented in this study are available from the corresponding author upon request.

## Supplementary Materials

The following are available online at https://www.mdpi.com/article/10.3390/insects13060505/s1, Table S1: Impact of clothianidin treatment on *L. decemlineata*.
