# Supplementary material for: Characterization of Insecticide Response-Associated Transcripts in the Colorado Potato Beetle: Relevance of Selected Cytochrome P450s and Clothianidin
Source: Insects. 2022 May 26;13(6):505. doi: 10.3390/insects13060505 (PMC9225154; doi:10.3390/insects13060505)
Supplement: Supplementary file 1 [file insects-13-00505-s001.zip › insects-1728190-supplementary.pdf]

Table S1.

Dose	Number of Insects (impaired/total)	Percentage
0.1 µg	2/5	40 %
0.4 µg	1/5	20 %
1.0 µg	4/5	80 %
2.5 µg	5/5	100 %
5.0 µg	5/5	100 %
10.0 µg	5/5	100 %
20.0 µg	5/5	100 %

Table S1. Impact of clothianidin treatment on *L. decemlineata*.
Presented are effects on insects exposed to varying doses of clothianidin.
